# Microbial pattern recognition suppresses *de novo* organogenesis

**DOI:** 10.1242/dev.201485

**Published:** 2023-05-03

**Authors:** Sorrel Tran, Yun-Fan Stephanie Chen, Dawei Xu, Madalene Ison, Li Yang

**Affiliations:** Department of Plant Pathology, College of Agricultural and Environmental Sciences, University of Georgia, Athens, GA 30602, USA

**Keywords:** *De novo* root regeneration, Microbial recognition, Regeneration, Salicylic acid

## Abstract

*De novo* root regeneration (DNRR) is a developmental process that regenerates adventitious roots from wounded tissues. Phytohormone signaling pathways involved in microbial resistance are mobilized after cutting and influence *de novo* root regeneration. Microbes may positively or negatively influence the development and stress responses of a plant. However, most studies on the molecular mechanisms of *de novo* organogenesis are performed in aseptic conditions. Thus, the potential crosstalk between organ regeneration and biotic stresses is underexplored. Here, we report the development of a versatile experimental system to study the impact of microbes on DNRR. Using this system, we found that bacteria inhibited root regeneration by activation of, but not limited to, pathogen-associated molecular pattern (PAMP)-triggered immunity. Sensing bacteria-derived flagellin 22 peptide (flg22) inhibited root regeneration by interfering with the formation of an auxin maximum at the wound site. This inhibition relies on the receptor complex that recognizes microbial patterns but may bypass the requirement of salicylic acid signaling.

## Teaser

**Summary:** An experimental system that supports *de novo* organogenesis in the presence of microbes reveals that recognition of microbial patterns inhibits wound-induced regeneration in plants.

## INTRODUCTION

The propagation and production of many fruits, vegetables and ornamental plants rely on various forms of regeneration, including *de novo* organogenesis, grafting, somatic embryogenesis or tissue culture (Ikeuchi et al., 2016). In natural conditions, regeneration occurs in the presence of microbes, including environmental microbes, the rhizosphere/phyllosphere microbiome and endophytes. However, most studies of plant regeneration are performed in aseptic conditions (Loyola-Vargas and Vázquez-Flota, 2006; Smith, 2012). Overgrowth of bacteria and fungi on regeneration media leads to the inhibition of organogenesis, abortion of plant tissues and other deleterious consequences. Microbes are often treated as contamination and eliminated from regeneration systems, so it is still unclear how the immune system governing plant biotic interactions influences the cell fate transition during regeneration.

As a part of their immune system, plants can recognize conserved molecules derived from microbial pathogens, i.e. pathogen/microbe-associated molecular patterns (PAMPs/MAMPs), through surface-localized pattern recognition receptors (PRRs) (Zhang et al., 2010). In *Arabidopsis*, these PRRs include FLAGELLIN-SENSITIVE 2 (FLS2, which recognizes flagellin), EF-TU RECEPTOR (EFR, which recognizes EF-Tu) and CHITIN ELICITOR RECEPTOR KINASE 1 (CERK1, which recognizes chitin) (Miya et al., 2007; Zipfel et al., 2004, 2006)*.* Loss-of-function mutations in these genes enhance susceptibility to various pathogens, such as the bacterial pathogen *P. syringae* pv. *tomato* (*Pst*) DC3000 (Zipfel et al., 2004, 2006; Willmann et al., 2011). PAMP perception triggers a signaling cascade that collectively results in a pattern-triggered immunity (PTI) response (DeFalco and Zipfel, 2021). PTI induces the activation of MAPK cascades, upregulation of defense genes, production of a hydrogen peroxide oxidative burst, callose deposition and enhanced pathogen resistance (DeFalco and Zipfel, 2021). Full activation of PTI requires co-receptors, such as BRI1-ASSOCIATED RECEPTOR KINASE (BAK1), and other cytoplasmic receptor-like kinases, including BAK1-LIKE 1 (BKK1) (Chinchilla et al., 2009; Roux et al., 2011; Sun et al., 2013). As BAK1 and BKK1 also interact with other PRRs, the *bak1-5/bkk1-1/cerk1* (*bbc*) triple mutant is compromised in defense signaling downstream of the recognition of multiple PAMPs (Xin et al., 2016).

Transcriptional signatures against microbial pathogens are activated during *de novo* organogenesis performed in aseptic conditions (Ikeuchi et al., 2017; Zhang et al., 2019; Liu et al., 2022). For example, responses to chitin, responses to salicylic acid (SA) and a defense response to fungi are activated after cutting a hypocotyl (Ikeuchi et al., 2017), despite the lack of elicitors in the experimental system. The activation of chitin and SA responses are controlled by WOUND INDUCED DEDIFFERENTIATION 1 (WIND1), a key regulator of wound-induced regeneration (Ikeuchi et al., 2017). Defense-related genes are also induced at an early stage of *de novo* root regeneration (DNRR), a process producing adventitious roots from leaf or stem cuttings (Hernandez-Coronado et al., 2022). In *Arabidopsis*, the SA response is induced a few hours after cutting (Liu et al., 2022) and can last up to 1 day after cutting (Hernandez-Coronado et al., 2022; Zhang et al., 2019). Functionally, SA represses various forms of regeneration acting downstream of glutamate receptors (Hernandez-Coronado et al., 2022; Tran et al., 2023). Mutants defective in SA biosynthesis or signaling enhance callus formation and adventitious root generation (Hernandez-Coronado et al., 2022; Tran et al., 2023). However, it is unclear how these defense-related responses may contribute to tissue regeneration in the presence of microbes.

Here, we report an experimental system that can support DNRR in the presence of microbes. We found that pathogenic bacteria and their disarmed mutants inhibited DNRR, whereas *E.coli* did not alter the regeneration capacity. The application of microbes suppressed DNRR, which was associated with a lack of auxin maximum in converter cells and compromised activation of cell fate transition markers. Flg22-mediated suppression required FLS2 and its co-receptors, but not endogenous SA. In addition, a soil bacterial community inhibited root regeneration, bypassing the requirement of PTI machinery and SA signaling. In summary, our experimental system provides a platform to study the link between biotic stresses and tissue regeneration.

## RESULTS AND DISCUSSION

### Sand plates supported DNRR

**Fig. 1.**
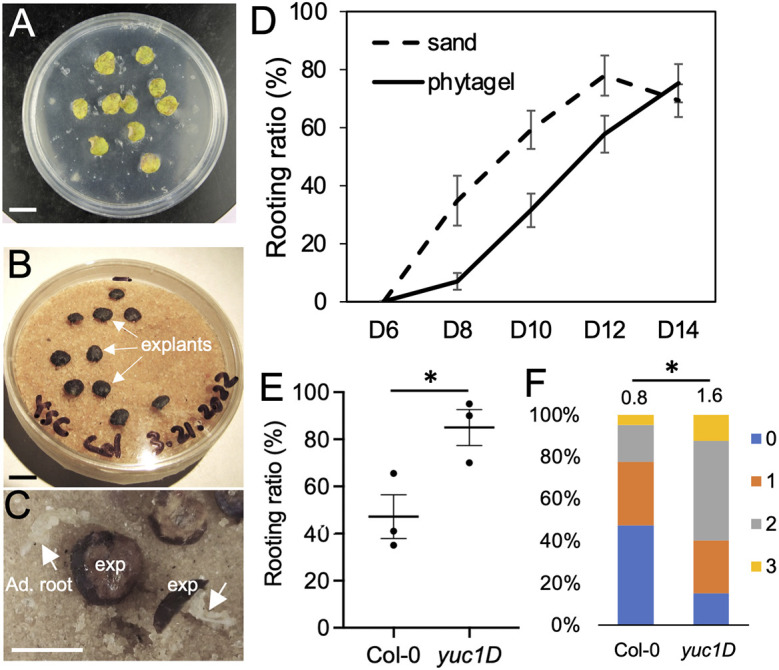
**Sand plates supported DNRR.** (A) Image of explants on an agar plate contaminated with bacteria. (B) Explants on a sand plate at 10 DAC. (C) Adventitious roots (arrows) formed at cutting site of explants on sand plates. (D) Comparison of rooting ratio between Col-0 explants on phytagel plates and sand plates. Data are mean±s.e.m. (E) Rooting ratio of Col-0 and *yuc1D* at 10 DAC on sand plates, each dot represents the rooting ratio of an independent experiment with 20 explants. *P<0.01 (unpaired two-tailed Student's *t*-test). Data are mean±s.e.m. (F) A comparison of adventitious root number formed on Col-0 and *yuc1D* on sand plates. Numbers indicate the number of adventitious roots from each explant. *P<0.01 (Mann–Whitney test). Scale bars: 1 cm in A,B; 5 mm in C.

In a regular DNRR assay, explants cut from the first two leaves of a 12-day-old seedling were placed on 1% agar or 0.3% phytagel containing B5 medium (Chen et al., 2014). Microbial contamination, either by a single bacterial strain (e.g. *Escherichia coli* DH5α) or by bacterial communities extracted from soil, led to explant abortion before setting roots (Fig. 1A). During this process, bacteria quickly multiplied on media, formed a bacterial lawn and covered explants (Fig. 1A). Thus, microorganisms are often treated as contamination that threatens the regeneration process and are eliminated in studies of DNRR. To develop an experimental system supporting the study of the microbial impact on DNRR, we tested several supporting media, including plain agar, autoclaved soil, filter paper and autoclaved sand. Among these media, DNRR occurred on autoclaved sand supplemented with sterile water was comparable with that on phytagel plates (Fig. 1B,C). Explants from leaves 1 and 2 of ecotype Columbia-0 (Col-0) were placed on sand with the abaxial sides facing downward. Starting from 7 days after cutting (DAC), we observed adventitious root formation from the cutting sites. At 12 DAC, about 90% of explants on sand produced adventitious roots (Fig. 1D). Explants on sand plates generated roots earlier than those on phytagel plates (Fig. 1D). The average number of adventitious roots generated from each explant on sand was comparable with those on phytagel plates. We also noticed that sand composed of uniform small granules worked better than coarse sand. Previous studies performed on gels showed that explants from a dominant gain-of-function *yucca* mutant, *yuc1D*, had a higher rooting capacity due to elevated auxin level (Chen et al., 2016). We observed the same trend of rooting capacity from mutant explants on sand medium (Fig. 1E), indicating that the mechanisms governing DNRR on agar/phytagel plates are comparable with those on sand plates. Explants from y*uc1D* also produced more adventitious roots than those from Col-0 on sand plates (Fig. 1F). These results indicate that sand could support DNRR from leaf explants.

### Single bacteria strains influence DNRR differently

**Fig. 2.**
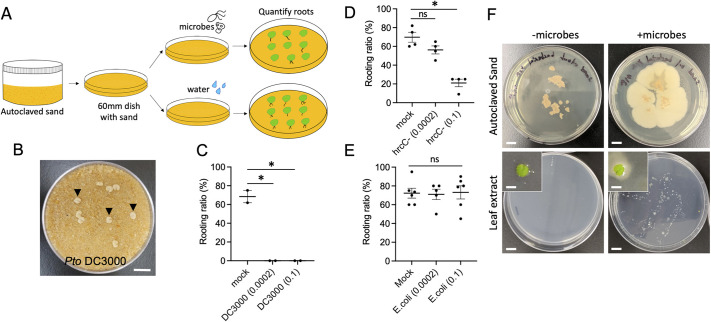
**Different bacteria have a distinct impact on DNRR on sand plates.** (A) The workflow to use sand as a medium to study DNRR under biotic interactions (for details, see Material sand Methods). (B) Bleached explants on a sand plate with *Pto* DC3000. (C-E) Rooting ratio of explants on sand plates when inoculated with *Pto* DC3000 (C), hrcC− (D) and *E.coli* (E). Each dot represents the rooting ratio of an independent experiment with 20 explants. *P<0.01 (unpaired two-tailed Student's *t*-test); ns, not significant. Data are mean±s.e.m. (F) A representative image of explants and sand samples collected after counting roots. Sand samples were directly moved from rooting plates to LB plates. Explants were homogenized in 1 ml sterile water. 10 µl of suspension were plated on LB plates. Bacteria were only detected in sand and in explants from inoculated plates. Scale bars: 1 cm in B and F (main panels); 5 mm in F (insets).

Among all the media tested, sand plates supported DNRR in the presence of bacteria (Fig. 2). To test how bacteria influence DNRR, we challenged explants on sand plates with a single bacteria strain of *Pto* DC3000 (a model bacterial pathogen of *Arabidopsis*), hrcC− (a mutant of *Pto* DC3000 defective in effector delivery) or *E. coli* DH5α (a commensal bacterium) (Fig. 2). A bacterial suspension in water was added to autoclaved sand before explants were placed on sand. We did not observe the formation of a bacterial colony or lawn on sand medium or on explants. When bacteria were added at a concentration of OD_600_=0.1 or 0.0002 (estimated from a 1:500 dilution of OD_600_=0.1), pathogenic *Pto* DC3000 killed all explants within 10 days (Fig. 2B), and no adventitious roots were formed on explants (Fig. 2C). The aborted explants were all bleached (Fig. 2B), which was different from the chlorosis and necrosis symptoms that developed on leaves attached to shoots (Mittal and Davis, 1995; Cameron et al., 1994). With the hrcC− treatment, most explants survived to 16 DAC on sand plates. At the high concentration (OD_600_=0.1), hrcC− reduced adventitious root formation from 75% to 17% (Fig. 2D). At a lower concentration (OD_600_=0.0002), hrcC− slightly reduced the rooting ratio and number of adventitious roots per explant, indicating a concentration-dependent effect on suppressing DNRR (Fig. 2D). *E.coli* did not alter rooting capacity of explants at either a low or high concentration (Fig. 2E). It is noteworthy that the amount of bacterial suspension should not exceed the surface of sand, because excessive liquid may lead to explant abortion. At the end of each experiment, we collected explants and sand from those plates and found that bacteria were present in both samples, indicating that the root regeneration occurred in the presence of bacteria (Fig. 2F). These observations demonstrated that the sand plates could support DNRR in the presence of different bacteria strains, and these bacteria had a distinct impact on DNRR.

### flg22 inhibited DNRR

**Fig. 3.**
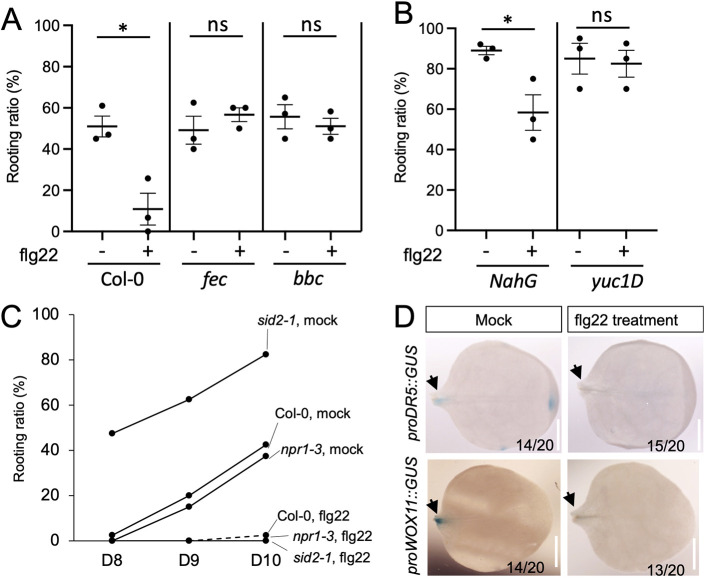
**Flg22 inhibited DNRR.** (A) Flg22-mediated inhibition of DNRR depended on FLS2 and co-receptors. Each dot represents the rooting ratio of an independent experiment with 20 explants. (B) Flg22-mediated inhibition of DNRR was compromised in *yuc1D*, but not *NahG*. *P<0.01 (unpaired two-tailed Student’ *t*-test); ns, not significant. Data are mean±s.e.m. Each dot represents the rooting ratio of an independent experiment with 20 explants. (C) Flg22 suppressed rooting capacity in *sid2-1* and *npr1-3* mutants. *n*=40. (D) Staining for GUS activity driven by the *DR5* and *WOX11* promoters in explants. DR5 promoter activity was evident at cutting sites at 2 DAC. The WOX11 promoter was activated at 4 DAC. Flg22 treatment reduced the GUS activity driven by both promoters. Arrows indicate the cutting sites. Scale bars: 2 mm.

We reasoned that activating PTI may contribute to the inhibition of DNRR. We tested whether flg22, a 22 amino acid peptide derived from bacterial flagellin, could inhibit DNRR. Explants were cut and cultured on aseptic phytagel plates. 12-day-old Col-0 explants were pre-treated with 1 µM of flg22 by spraying the seedlings 1 h before cutting. An additional 10 µl of flg22 was directly added to the cut site of each explant after placing explants on plate. At 10 DAC, the rooting ratio on control Col-0 explants was around 50%, with a 1 µM flg22 treatment significantly reducing the rooting ratio to 15% in Col-0 (Fig. 3A). In the *fls2/efr/cerk1* (*fec*) mutant (lacking multiple PRRs) and *bbc* mutant (lacking co-receptors), the flg22-induced suppression was not observed, demonstrating that the suppression required the same upstream components involved in flg22-induced PTI (Fig. 3A).

SA signaling is often activated after perceiving microbial signals, which contribute to the full activation of PTI (Seyfferth and Tsuda, 2014; Bigeard et al., 2015). Activating SA signaling also inhibited various forms of regeneration, including *de novo* organogenesis (Hernandez-Coronado et al., 2022). To test the hypothesis that SA signaling downstream of flg22-induced PTI suppressed rooting, we tested the rooting capacity of *NahG* (a transgenic plant expressing a SA hydroxylase from the bacterium *Pseudomonas putida*) under mock and flg22 treatments. Despite the high rooting level of *NahG*, flg22 suppressed rooting in *NahG* explants to a similar extent to the expression in Col-0 (Fig. 3B). Consistently, flg22 treatment inhibited rooting from *sid2-1* and *npr1-3* explants (Fig. 3C). *Sid2-1* carries a mutation in *Arabidopsis* ISOCHORISMATE SYNTHASE 1 and is defective in SA accumulation, whereas *npr1-3* is a loss-of-function mutant of key SA receptor for immune signaling. The DNRR phenotypes of *sid2-1* and *npr1-3* on sand plates were consistent with their phenotypes on phytagel plates (Tran et al., 2023). These observations indicate that SA was not the sole contributor of flg22-mediated inhibition of DNRR. Flg22-induced Ca^2+^ influx, a reactive oxygen species (ROS) burst or the MAP kinase cascade may also suppress regeneration (Bigeard et al., 2015; DeFalco and Zipfel, 2021).

As flg22 treatment can inhibit root elongation (Gómez-Gómez and Boller, 2000), we further monitored the activation of *WUSCHEL RELATED HOMEOBOX11* (*WOX11*), a marker of cell fate transition in converter cells (Liu et al., 2014). Promoter activity of *WOX11* was activated at 2 DAC at the wound site (Liu et al., 2014). Flg22 treatment dramatically inhibited the expression of *WOX11*, suggesting that the reduced rooting was due to disrupted cell fate transition rather than to root elongation (Fig. 3D). We further examined the expression of *proDR5::GUS* in mock and flg22-treated explants because high levels of auxin accumulation at the cutting site are responsible for the activation of *WOX11* (Fig. 3D). We found that *proDR5::GUS* activity was inhibited by flg22 at the wound site, indicating that flg22 may repress DNRR by interfering with auxin signaling (Fig. 3D). Consistent with its impact on the *proDR5::GUS* activity, flg22-mediated suppression of DNRR was blocked in *yuc1D* mutant (Fig. 3B). Taken together, flg22 may act upstream of auxin biosynthesis to suppress DNRR.

### The soil bacteria community inhibited DNRR

**Fig. 4.**
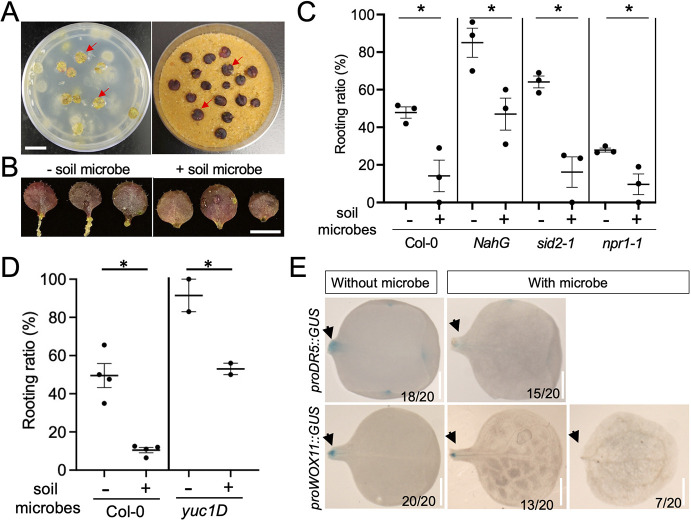
**Soil microbes inhibited DNRR.** (A) Images of a phytagel plate (left) and a sand plate (right) inoculated with a soil bacterial community. Red arrows indicate the explants. All explants on the sand plate survived; no bacterial lawn was observed. (B) Images of explants from sand plates with or without soil microbes. (C) The soil bacteria community inhibited DNRR in Col-0, *NahG*, *sid2-1* and *npr1-1* mutants. (D) The soil bacteria community inhibited rooting in *yuc1D*. Statistics comparing mock and soil bacterial treatment were carried out using an unpaired two-tailed Student's *t*-test. *P<0.01; ns, not significant. Data are mean±s.e.m. Each dot represents the rooting ratio of an independent experiment with 20 explants. (E) Staining for GUS activity driven by *DR5* and *WOX11* promoter in explants. Soil bacteria inhibited the *DR5* and *WOX11* promoter activity. The staining for *DR5* and *WOX11* was performed at 2 DAC and 4 DAC, respectively. Arrows indicate the cutting sites. Scale bars: 1 cm in A; 5 mm in B; 2 mm in E.

To test the capacity of sand plates to handle a complex bacterial community, we supplemented sand plates with a soil bacteria community extracted from potting soil (Sungro Propagation Mix). Unlike the overgrowth of bacteria on a phytagel plate, no bacterial colonies or lawn were observed within 12 DAC on a sand plate (Fig. 4A). Bacterial treatment significantly reduced the adventitious root formation, but the explants were still alive and otherwise comparable with the non-treated explants (Fig. 4B). At the end of this experiment, we recovered a diversified bacterial community from sand and explants. At 10 DAC, explants on control sand plates had, on average, a 56% rooting ratio, while on sand plates with bacteria the ratio was 15% (Fig. 4C). Soil microbes also suppressed DNRR in SA accumulation (*NahG* and *sid2-1*) and signaling (*npr1-1*) mutants (Fig. 4C). Unlike flg22, soil microbes reduced rooting capacity in the *yuc1D* mutant (Fig. 4D), indicating that members in the community may suppress regeneration downstream of auxin accumulation. Microbe treatment also suppressed the activity of *proDR5::GUS* and *proWOX11::GUS* at the cutting site (Fig. 4E), implying that a soil bacteria community may inhibit DNRR through multiple signaling events. Bacteria widely influence phytohormone crosstalk by producing hormone mimics or by interfering with hormone signaling. It is possible that some members in the soil bacteria community suppressed DNRR by modulating other hormone pathways, such as jasmonic acid (JA) or auxin (Egamberdieva et al., 2017; Kazan and Lyons, 2014; Costacurta and Vanderleyden, 1995).

In summary, we report an experimental system that allows the study of *de novo* root regeneration under biotic stresses. We show that activating PTI suppresses DNRR, but other signaling pathways may inhibit DNRR via a PTI-independent manner when specific bacterial strains or a soil bacteria community are present. Our research offers a tool for dissecting the potential impact of environmental microbes or endophytes on cell fate specification during tissue regeneration.

## MATERIALS AND METHODS

### DNRR assay on phytagel plates

*Arabidopsis* seeds were sterilized in 70% ethanol for 10 min and planted onto ½ MS medium. After 2 days of incubation at 4°C, seeds were moved to continuous light conditions at 23°C. The 1st and 2nd true leaves from 12-day-old seedlings were cut at the junction between the leaf blade and petiole, and placed with the abaxial side down onto Gamborg's B5 media (RPI Research Products International) with 0.5 g/L MES (VWR Life Sciences) and 0.3% w/v phytagel (MilliporeSigma) at pH 5.7. Each plate contained 12 ml of B5 media and 20 leaf explants.

### A DNRR assay on sand plates

Autoclaved play sand (27 g; Quikrete) was added to a 60 mm×15 mm Petri dish. 7 ml of autoclaved water or 7 ml of microbe resuspension in water was added to each sand plate as a mock or microbe treatment. Each plate holds about 20 explants. Plates were sealed with Micropore tape (3 M) and placed in a growth chamber (Percival Scientific) with continuous light at 23°C. Plates were opened on the indicated DAC for root counting. Sample sand and explants were collected after counting and placed on LB medium to confirm the presence or absence of bacteria at the end point of each experiment. Opened plates were discarded.

### Microbe treatment on explants

For single strain treatment, overnight bacterial culture was precipitated and re-suspended in water. 7 ml of bacteria suspension at the indicated OD was added into each 27 g of autoclaved sand in a 60 mm×15 mm Petri dish. Explants were directly placed onto inoculated sand.

For soil microbe treatment, approximately 3 g of potting soil was mixed and vortexed with 45 ml of water. After filtering through a cotton ball, the suspension was centrifuged for 5 min at 7000 ***g*** in a refrigerated centrifuge. Liquid was discarded and pellets were re-suspended in water. The suspension was filtered through a 0.45 µm syringe filter and was used as soil bacteria mixture. The final OD_600_ is about 0.07. 7 ml of soil bacterial mixture was mixed with sand before explants were placed on plates.

### Flg22 treatment

Flg22 stock (1 mM) in DMSO was diluted with sterile water to final concentrations at 1 µM. The Flg22 sequence is QRLSTGSRINSAKDDAAGLQIA. A 1000× dilution of DMSO was used as mock control. 12-day-old plants were pre-treated with flg22 or mock for 1 h. Leaf explants were placed on B5 medium [Gamborg B5 basal medium with 0.5 g/L MES, 0.8% agar (pH 5.7)]. 10 µl of flg22 was also added to the cutting sites.

### GUS staining

The explants were submerged in a GUS staining solution [100 mM sodium phosphate (pH 7.0), 1 mM EDTA (pH 8), 1% Triton-X-100, 5 mM potassium ferrocyanide, 5 mM potassium ferricyanide and 1 mg/ml X-Gluc] and were vacuum infiltrated for 10 min and incubated at 37°C overnight. After staining, leaf explants were washed with increasing concentrations of ethanol (15%, 50% and 70%) for 1 h, fixed in 70% ethanol for 48 h and washed with 0.1 M phosphate buffer (pH 6.8). Leaf explants were mounted on slides using ClearSee solution (Kurihara et al., 2015) and then imaged under a VWR Stereo zoom trinocular microscope equipped with a VWR digital microscope camera.

## References

[DEV201485C1] Bigeard, J., Colcombet, J. and Hirt, H. (2015). Signaling mechanisms in pattern-triggered immunity (PTI). *Mol. Plant* 8, 521-539. 10.1016/j.molp.2014.12.022 25744358

[DEV201485C2] Cameron, R. K., Dixon, R. A. and Lamb, C. J. (1994). Biologically induced systemic acquired resistance in Arabidopsis thaliana. *Plant J.* 5, 715-725. 10.1111/j.1365-313X.1994.00715.x

[DEV201485C3] Chen, X., Qu, Y., Sheng, L., Liu, J., Huang, H. and Xu, L. (2014). A simple method suitable to study de novo root organogenesis. *Front. Plant Sci.* 5, 208. 10.3389/fpls.2014.00208 24860589 PMC4030142

[DEV201485C4] Chen, L., Tong, J., Xiao, L., Ruan, Y., Liu, J., Zeng, M., Huang, H., Wang, J.-W. and Xu, L. (2016). YUCCA-mediated auxin biogenesis is required for cell fate transition occurring during de novo root organogenesis in Arabidopsis. *J. Exp. Bot..* 67, 4273-4284. 10.1093/jxb/erw213 27255928 PMC5301932

[DEV201485C5] Chinchilla, D., Shan, L., He, P., De Vries, S. and Kemmerling, B. (2009). One for all: the receptor-associated kinase BAK1. *Trends Plant Sci.* 14, 535-541. 10.1016/j.tplants.2009.08.002 19748302 PMC4391746

[DEV201485C6] Costacurta, A. and Vanderleyden, J. (1995). Synthesis of phytohormones by plant-associated bacteria. *Crit. Rev. Microbiol.* 21, 1-18. 10.3109/10408419509113531 7576148

[DEV201485C7] Defalco, T. A. and Zipfel, C. (2021). Molecular mechanisms of early plant pattern-triggered immune signaling. *Mol. Cell.* 81, 3449-3467. 10.1016/j.molcel.2021.07.029 34403694

[DEV201485C8] Egamberdieva, D., Wirth, S. J., Alqarawi, A. A., Abd Allah, E. F. and Hashem, A. (2017). Phytohormones and beneficial microbes: essential components for plants to balance stress and fitness. *Front. Microbiol.* 8, 2104. 10.3389/fmicb.2017.02104 29163398 PMC5671593

[DEV201485C9] Gómez-Gómez, L. and Boller, T. (2000). FLS2: an LRR receptor–like kinase involved in the perception of the bacterial elicitor flagellin in Arabidopsis. *Mol. Cell.* 5, 1003-1011. 10.1016/S1097-2765(00)80265-8 10911994

[DEV201485C10] Hernandez-Coronado, M., Dias Araujo, P. C., Ip, P. L., Nunes, C. O., Rahni, R., Wudick, M. M., Lizzio, M. A., Feijo, J. A. and Birnbaum, K. D. (2022). Plant glutamate receptors mediate a bet-hedging strategy between regeneration and defense. *Dev. Cell.* 57, 451-465.e6. 10.1016/j.devcel.2022.01.013 35148835 PMC8891089

[DEV201485C11] Ikeuchi, M., Ogawa, Y., Iwase, A. and Sugimoto, K. (2016). Plant regeneration: cellular origins and molecular mechanisms. *Development* 143, 1442-1451. 10.1242/dev.134668 27143753

[DEV201485C12] Ikeuchi, M., Iwase, A., Rymen, B., Lambolez, A., Kojima, M., Takebayashi, Y., Heyman, J., Watanabe, S., Seo, M., De Veylder, L. et al. (2017). Wounding triggers callus formation via dynamic hormonal and transcriptional changes. *Plant Physiol.* 175, 1158-1174. 10.1104/pp.17.01035 28904073 PMC5664475

[DEV201485C13] Kazan, K. and Lyons, R. (2014). Intervention of phytohormone pathways by pathogen effectors. *Plant Cell.* 26, 2285-2309. 10.1105/tpc.114.125419 24920334 PMC4114936

[DEV201485C14] Kurihara, D., Mizuta, Y., Sato, Y. and Higashiyama, T. (2015). ClearSee: a rapid optical clearing reagent for whole-plant fluorescence imaging. *Development* 142, 4168-4179. 10.1242/dev.127613 26493404 PMC4712841

[DEV201485C15] Liu, J., Sheng, L., Xu, Y., Li, J., Yang, Z., Huang, H. and Xu, L. (2014). WOX11 and 12 are involved in the first-step cell fate transition during de novo root organogenesis in Arabidopsis. *Plant Cell.* 26, 1081-1093. 10.1105/tpc.114.122887 24642937 PMC4001370

[DEV201485C16] Liu, W., Zhang, Y., Fang, X., Tran, S., Zhai, N., Yang, Z., Guo, F., Chen, L., Yu, J. and Ison, M. S. (2022). Transcriptional landscapes of de novo root regeneration from detached Arabidopsis leaves revealed by time-lapse and single-cell RNA sequencing analyses. *Plant Commun.* 3, 100306. 10.1016/j.xplc.2022.100306 35605192 PMC9284295

[DEV201485C17] Loyola-Vargas, V. M. and Vázquez-Flota, F. (2006). *Plant Cell Culture Protocols*. Springer. 10.1385/1-59259-959-1:003 16673901

[DEV201485C18] Mittal, S. and Davis, K. R. (1995). Role of the phytotoxin coronatine in the infection of Arabidopsis thaliana by Pseudomonas syringae pv. tomato. *Mol. Plant Microbe Interact.* 8, 165-171. 7539639 10.1094/mpmi-8-0165

[DEV201485C19] Miya, A., Albert, P., Shinya, T., Desaki, Y., Ichimura, K., Shirasu, K., Narusaka, Y., Kawakami, N., Kaku, H. and Shibuya, N. (2007). CERK1, a LysM receptor kinase, is essential for chitin elicitor signaling in Arabidopsis. *Proc. Natl. Acad. Sci. USA* 104, 19613-19618. 18042724 10.1073/pnas.0705147104 PMC2148337

[DEV201485C20] Roux, M., Schwessinger, B., Albrecht, C., Chinchilla, D., Jones, A., Holton, N., Malinovsky, F. G., Tör, M., De Vries, S. and Zipfel, C. (2011). The Arabidopsis leucine-rich repeat receptor–like kinases BAK1/SERK3 and BKK1/SERK4 are required for innate immunity to hemibiotrophic and biotrophic pathogens. *Plant Cell.* 23, 2440-2455. 10.1105/tpc.111.084301 21693696 PMC3160018

[DEV201485C21] Seyfferth, C. and Tsuda, K. (2014). Salicylic acid signal transduction: the initiation of biosynthesis, perception and transcriptional reprogramming. *Front. Plant Sci.* 5, 697. 10.3389/fpls.2014.00697 25538725 PMC4260477

[DEV201485C22] Smith, R. H. (2012). *Plant Tissue Culture: Techniques and Experiments*. Academic Press.

[DEV201485C23] Sun, Y., Li, L., Macho, A. P., Han, Z., Hu, Z., Zipfel, C., Zhou, J.-M. and Chai, J. (2013). Structural basis for flg22-induced activation of the Arabidopsis FLS2-BAK1 immune complex. *Science* 342, 624-628. 10.1126/science.1243825 24114786

[DEV201485C24] Tran, S., Ison, M., Ferreira Dias, N. C., Ortega, M. A., Chen, Y. S., Peper, A., Hu, L., Xu, D., Mozaffari, K., Severns, P. M. et al. (2023). Endogenous salicylic acid suppresses de novo root regeneration from leaf explants. *PLoS Genet.* 19, e1010636. 10.1371/journal.pgen.1010636 36857386 PMC10010561

[DEV201485C25] Willmann, R., Lajunen, H. M., Erbs, G., Newman, M.-A., Kolb, D., Tsuda, K., Katagiri, F., Fliegmann, J., Bono, J.-J. and Cullimore, J. V. (2011). Arabidopsis lysin-motif proteins LYM1 LYM3 CERK1 mediate bacterial peptidoglycan sensing and immunity to bacterial infection. *Proc. Natl. Acad. Sci. USA* 108, 19824-19829. 22106285 10.1073/pnas.1112862108 PMC3241766

[DEV201485C26] Xin, X.-F., Nomura, K., Aung, K., Velásquez, A. C., Yao, J., Boutrot, F., Chang, J. H., Zipfel, C. and He, S. Y. (2016). Bacteria establish an aqueous living space in plants crucial for virulence. *Nature* 539, 524-529. 10.1038/nature20166 27882964 PMC5135018

[DEV201485C27] Zhang, Y., Yang, Y., Fang, B., Gannon, P., Ding, P., Li, X. and Zhang, Y. (2010). Arabidopsis snc2-1D activates receptor-like protein-mediated immunity transduced through WRKY70. *Plant Cell* 22, 3153-3163. 10.1105/tpc.110.074120 20841424 PMC2965549

[DEV201485C28] Zhang, G., Zhao, F., Chen, L., Pan, Y., Sun, L., Bao, N., Zhang, T., Cui, C.-X., Qiu, Z., Zhang, Y. et al. (2019). Jasmonate-mediated wound signalling promotes plant regeneration. *Nat. Plants* 5, 491-497. 10.1038/s41477-019-0408-x 31011153

[DEV201485C29] Zipfel, C., Robatzek, S., Navarro, L., Oakeley, E. J., Jones, J. D., Felix, G. and Boller, T. (2004). Bacterial disease resistance in Arabidopsis through flagellin perception. *Nature* 428, 764-767. 10.1038/nature02485 15085136

[DEV201485C30] Zipfel, C., Kunze, G., Chinchilla, D., Caniard, A., Jones, J. D., Boller, T. and Felix, G. (2006). Perception of the bacterial PAMP EF-Tu by the receptor EFR restricts Agrobacterium-mediated transformation. *Cell* 125, 749-760. 10.1016/j.cell.2006.03.037 16713565

