# Supplementary material for: Microbial pattern recognition suppresses de novo organogenesis
Source: Development. 2023 May 3;150(9):dev201485. doi: 10.1242/dev.201485 (PMC10259513; doi:10.1242/dev.201485)
Supplement: Supplementary file 1 [file develop-150-201485_review_history.pdf]

Development

Microbial pattern recognition suppresses *de novo* organogenesis

Sorrel Tran, Yun-Fan Stephanie Chen, Dawei Xu, Madalene Ison and Li Yang
DOI: 10.1242/dev.201485

Editor: Ykä Helariutta

Review timeline

Original submission:	25 November 2022
Editorial decision:	9 January 2023
First revision received:	17 March 2023
Accepted:	3 April 2023

Original submission

First decision letter

MS ID#: DEVELOP/2022/201485

MS TITLE: Microbial Pattern Recognition suppresses de novo organogenesis

AUTHORS: Sorrel Tran, Yun Fan Stephanie Chen, Dawei Xu, Madalene Ison, and Li Yang

I have now received all the referee's reports on the above manuscript, and have reached a decision. The referee's comments are appended below, or you can access them online: please go to BenchPress and click on the 'Manuscripts with Decisions' queue in the Author Area.

As you will see, the referee expresses considerable interest in your work, but has some significant criticisms and recommends a substantial revision of your manuscript before we can consider publication. If you are able to revise the manuscript along the lines suggested, which may involve further experiments, I will be happy receive a revised version of the manuscript. Your revised paper will be re-reviewed by one or more of the original referees, and acceptance of your manuscript will depend on your addressing satisfactorily the reviewers' major concerns. Please also note that Development will normally permit only one round of major revision. If it would be helpful, you are welcome to contact us to discuss your revision in greater detail. Please send us a point-by-point response indicating your plans for addressing the referee's comments, and we will look over this and provide further guidance.

Please attend to all of the reviewer's comments and ensure that you clearly highlight all changes made in the revised manuscript. Please avoid using 'Tracked changes' in Word files as these are lost in PDF conversion. I should be grateful if you would also provide a point-by-point response detailing how you have dealt with the points raised by the reviewers in the 'Response to Reviewers' box. If you do not agree with any of their criticisms or suggestions please explain clearly why this is so.

Reviewer 1

Advance summary and potential significance to field

The ms by Tran et al. "Microbial Pattern Recognition suppresses de novo organogenesis" is a straightforward set of results addressing the basic question of the relationship two key plant responses: regeneration and pathogen response. Previous work has suggested a tradeoff between the two responses, but a direct test of the effect of pathogens on regeneration has not been tested. This paper is a logical follow up. One barrier to that follow up is that the systems we use to

© 2023. Published by The Company of Biologists under the terms of the Creative Commons Attribution License (https://creativecommons.org/licenses/by/4.0/).

test pathogen responses generally kill the tissue they infect on standard plate conditions. That may not be a realistic scenario for most plants that encounter many pathogens in the environment that do not necessarily immediately kill the plant tissue. The authors develop an important experimental system for de novo root regeneration on sand that represents a nice addition to the toolkit for testing plant responses to pathogens. They show nicely in this system that flg22 and soil bacteria elicit a pathogen response, without killing tissue, that inhibits regeneration. They further show that the pathway mediating the “tradeoff” is independent of salicylic acid.

Comments for the author

I think the paper is nicely done and I have just two major comments.

1. I am a bit surprised and not totally convinced that biotrophic response pathway through SA is not involved in the pathogen response that mediates the tradeoff with regeneration. The NahG system has been used before to test the role of the salicylic acid pathway, but even then it has been used in conjunction with salicylic acid response pathway mutants. The NPR1 mutant, for example, has been a powerful tool along those lines. The authors should try mutants in the pathway to back up that conclusion that the SA pathway is not involved. I think one other genetic test is needed to make the statement.

2. One convincing experiment was the use of soil bacteria to show that “natural” pathogens a plant is likely to encounter inhibit regeneration. Does the presence of soil bacteria inhibit growth? I don’t think that would change any of the conclusions but it could offer one important explanation for the decline in regeneration. I didn’t see that control experiment and, unless I missed it, that should be done.

Otherwise, I thought this was logical and nicely done experiment that addresses an important question left open by previous results. The development of the sand system showed good experimental strategy in realizing that common pathogen testing protocols do not represent realistic field conditions. I think both aspects of this short but well-executed manuscript will be important additions to the field.

First revision

Author response to reviewers' comments

1.I am a bit surprised and not totally convinced that biotrophic response pathway through SA is not involved in the pathogen response that mediates the tradeoff with regeneration. The NahG system has been used before to test the role of the salicylic acid pathway, but even then it has been used in conjunction with salicylic acid response pathway mutants. The NPR1 mutant, for example, has been a powerful tool along those lines. The authors should try mutants in the pathway to back up that conclusion that the SA pathway is not involved. I think one other genetic test is needed to make the statement.

Response:

We revised our statement in the abstract. In the original manuscript, we stated that “Such inhibition relied on the receptor complex recognizing microbial patterns but is independent of salicylic acid (SA) signaling”. Now, we revised it as “Such inhibition relied on the receptor complex recognizing microbial patterns but may bypass the requirement of salicylic acid (SA) signaling”.

In addition, we included new data supporting our conclusion. First, we show that flg22 inhibited DNRR in the sid2-1 mutant (defective in SA biogenesis) and npr1-3 (SA signaling mutant) (Figure 3C). Second, in new Figure 4C, we show that soil microbe community inhibited DNRR in NahG, sid2-1 and npr1-1.

We also included a discussion sentence to explain the SA-independent suppression. “flg22-induced Ca^{2+} influx, reactive oxygen species (ROS) burst, or MAP kinase cascade may also suppress regeneration.”

© 2023. Published by The Company of Biologists under the terms of the Creative Commons Attribution License (https://creativecommons.org/licenses/by/4.0/).

2.One convincing experiment was the use of soil bacteria to show that “natural” pathogens a plant is likely to encounter inhibit regeneration. Does the presence of soil bacteria inhibit growth? I don’t think that would change any of the conclusions but it could offer one important explanation for the decline in regeneration. I didn’t see that control experiment and, unless I missed it, that should be done.

Response:

In the revision, we showed that the presence of soil bacteria did not change biomass when seedlings were grown on sand plates (see attached figures). We measured average weight seedlings grown on sand plates with or without soil microbes, and no significant difference was observed.

In addition, we included a new figure (Figure 4B) comparing 10 DAC explants from sand plates with or without microbes. Despite the defects in generating adventitious root, the explants from microbe-containing plates were comparable to those on sterile plates.

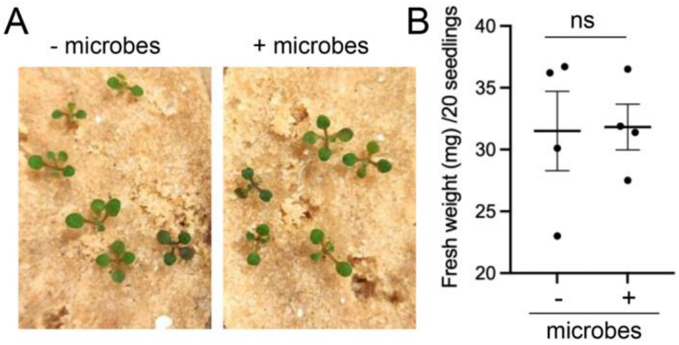

A: images of seedlings grown on sand plates with or without soil microbes. **B**: the average weight of seedlings was not different from two conditions. ns: not significant based on student t-test.

Second decision letter

MS ID#: DEVELOP/2022/201485

MS TITLE: Microbial Pattern Recognition suppresses de novo organogenesis

AUTHORS: Sorrel Tran, Yun Fan Stephanie Chen, Dawei Xu, Madalene Ison, and Li Yang
ARTICLE TYPE: Research Report

I am happy to tell you that your manuscript has been accepted for publication in Development, pending our standard ethics checks.

Reviewer 1

Advance summary and potential significance to field

The paper addresses a critical question about trade offs that the plant must deal with in the course of its life cycle. Here, the authors show that immune response effectors or bacteria inoculation compromise de novo root regeneration. In contrast to previous reports, they show this effect is independent of the SA pathway. This illustrates an important mechanism by which the plant regulates it's tradeoffs and defends against pathogen attacks.

Comments for the author

The author's have addressed my concerns with two clear experiments, using the npr1-3 (putative null) to test the independence of flg or bacterial induced inhibition of regeneration. The experiment seems to provide a definitive answer that the effect does not work through the SA pathway. In addition, they have supplied another critical control. The paper is nicely done and clearly written. I have no other concerns and I think this will make an important contribution to the regeneration and immunology fields.

© 2023. Published by The Company of Biologists under the terms of the Creative Commons Attribution License (https://creativecommons.org/licenses/by/4.0/).
